# Physicians’ Workloads in China: 1998–2016

**DOI:** 10.3390/ijerph15081649

**Published:** 2018-08-03

**Authors:** Yanhong Fu, David C. Schwebel, Guoqing Hu

**Affiliations:** 1 Department of Epidemiology and Health Statistics, Xiangya School of Public Health, Central South University, Changsha 410078, China; 176911022@csu.edu.cn; 2 Department of Psychology, University of Alabama at Birmingham, Birmingham, AL 35294 USA; schwebel@uab.edu

**Keywords:** physicians, workloads, health institutions

## Abstract

Background: Physicians play a primary role in patients’ health. Heavy workloads can threaten the health of physicians and their patients. This study examined workload changes among physicians in Chinese health institutions from 1998–2016. Methods: This study examined data from the online China Statistical Yearbook of 1999–2017, which is released annually by the National Bureau of Statistics of the People’s Republic of China. Three relevant and available indicators were retrieved: (1) number of physicians, (2) number of patient visits and (3) number of inpatient admissions. Patient visits per physician and inpatient admissions per physician from 1998–2016 were calculated to approximate physician workloads in Chinese health institutions. Results: Between 1998 and 2016, patient visits per physician in China increased by 135% and inpatient admissions per physician rose by 184%. Both indicators demonstrate a stabilizing trend in the most recent five years, including a slight decrease (7%) in patient visits per physician since 2012. Conclusions: Physician workload increased dramatically for Chinese physicians from 1998–2016, a trend that could potentially threaten physicians’ health and the quality of patient care. The findings highlight the importance of interventions and efforts to relieve physician workloads in China.

## 1. Introduction

Since the mid-1990s, China has initiated multiple health reforms to improve the quality of health service and increase equity in health service supply and utilization across the country. These reforms include improving the public health service network by enhancing basic functions, improving the medical service system, accelerating the construction of health care security systems and improving the drug supply system [1]. In the process of these reforms, physicians, some of the major providers of health services, may have been asked to shoulder more and more responsibilities for patient care, creating the possibility that physician workloads in China have increased dramatically over the past two decades. In fact, a 2014 survey conducted by the Chinese Medical Doctor Association reported that over 32% of physicians worked more than 60 h per week in China [2], violating legal limits for a 44-h work week in China. If true, heavy physician workloads could impact health for both the physicians themselves and the quality of health service they provide to their patients. Physicians could suffer from stress and exhaustion, ultimately leading to negative personal health outcomes and even death [3,4]. Physicians being overwork also can impact the quality of patient care, leading to poor or rushed doctor-patient communication, medical mistakes and poor patient satisfaction ratings [5,6,7]. Perhaps as a result of poor doctor-patient communication and heavy and stressful physician workloads, there are multiple reports of serious violence by patients against Chinese physicians [8,9].

Policy makers and hospital administrators may be unaware or ignorant of the impact of overworked physicians in China. This study used three national indicators to examine the extent of being overworked among Chinese physicians and the changes in physician workloads over recent years.

## 2. Materials and Methods

Data were extracted from the online China Statistical Yearbook of 1999–2017 [10], which is released annually by the National Bureau of Statistics of the People’s Republic of China. Because official statistics do not provide indicators that directly measure physicians’ workloads and related health outcomes, three available indicators were retrieved: (1) number of physicians, (2) number of patient visits and (3) number of inpatient admissions. Based on these three indicators, patient visits per physician and inpatient admissions per physician from 1998–2016 were calculated to approximate Chinese physician workloads in health institutions. Percent change in workload per physician between 1998 and 2016 was calculated as “(indicator in 2016–indicator in 1998)/indicator in 1998 * 100%”. Data analyses were performed through Stata Version 12.1 (StataCorp LLC, College Station, TX, USA) in May 2018.

## 3. Results

**Figure 1:**
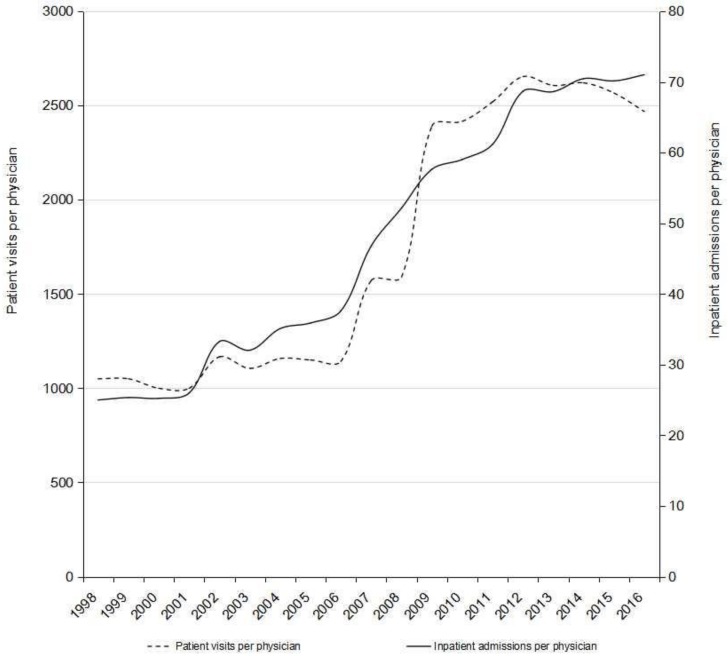
Patient visits and inpatients served by physicians on average in China, 1998–2016.

From 1998–2016, the number of physicians in China increased by 60% (from 2.0–3.2 million), but the number of patient visits attended by physicians and the number of inpatient admissions increased more dramatically, by 276% (from 2.1–7.9 billion) and 355% (from 50.0–227.3 million), respectively (Table 1). As a result, patient visits per physician increased by 135% (from 1050–2469 annually on average) and inpatient admissions per physician rose by 184% (from 25–71 annually on average) between 1998 and 2016. Both indicators demonstrate a stabilizing trend in the most recent five years, including a slight decrease (7%) in patient visits per physician since 2012 (Figure 1).

## 4. Discussion

Physician workloads have increased dramatically in China from 1998–2016. Although trends suggest a leveling of the growth over the past five years, physicians in 2016 were treating over three-times as many patients as physicians in 1998.

The rise in physician workloads is likely due primarily to government-driven health care reforms that led to a jump in health service utilization, which experienced 2.8- and 3.6-fold increases in patient visits and inpatient admissions respectively between 1998 and 2016. The physician workloads may be particularly heavy in larger urban hospitals since patients disproportionally flow to larger hospitals, although both central and local governments have taken some action to transfer patients from larger hospitals to smaller hospitals and community health centers [11].

The overall picture from the study’s findings is encouraging for public health in China. Expansion of basic social medical insurance coverage and reimbursement amounts since the early 2000s have produced greater equity in access to health care and improved health among poorer and rural populations [12,13]. However, the changes also appear to have created substantial increases in Chinese physician workloads. Over time, heavy physician workloads could threaten the health service system of China. Physicians could become exhausted and suffer health problems themselves, and quality of patient service could deteriorate.

In fact, a recent systematic review and meta-analysis by Kivimäki et al. concluded that overwork was associated with an increase in the risk of incident coronary heart disease (relative risk: 1.13, 95% confidence interval, 1.02–1.26) and incident stroke (relative risk: 1.33, 95% confidence interval, 1.11–1.61) [14]. Based on reports in a combination of local media, medical and news websites, official documents and scholarly articles, Shan et al. reported that the number of ‘Karoshi’(occupationally-related sudden death) increased from six in 2013 to 24 in 2015 among Chinese physicians [15].

Physician overwork also is documented to impact patient care. A multi-center cross-sectional survey of 1537 physicians at 46 hospitals across China showed that physicians working over 45 h per week were significantly more likely to commit medical mistakes compared to those working less than 45 h per week [7]. In addition, overwork has been demonstrated to lead to inferior doctor-patient communication, a factor that can impact patient health and lead in rare cases to violence against physicians [16,17].

This study was limited by including only three indicators to gauge study outcomes. Future research might include other variables that were not available in the datasets used for this study, including the workloads of other health professionals beyond physicians (nurses, physical therapists, etc.); physician workloads divided by health specialty (oncology, surgery, family medicine, etc.) and case severity; type of health service institution that physicians work in (hospital, primary care, public health institution, etc.); level of health service institution that physicians work in (national, provincial, municipal, county-level, town-level); and ownership of health service institutions (public, private). Future research might also consider the specific physician and patient health risks associated with increased physician workloads, perhaps through longitudinal methods that offer the opportunity to infer causality through an examination of trends over time.

## 5. Conclusions

Increased physician workloads in China merit the attention of researchers and policy-makers. To protect the health of its citizens, the government of China must protect the health of its physicians. Rigorous longitudinal studies should examine causal associations between increased physician workloads and both physician and patient health. Systematic interventions should be considered to relieve the heavy workloads that physicians face. These efforts could include increasing the number of hospitals and properly-trained physicians; transferring patients when possible from larger urban hospitals to less burdened smaller hospitals and community health centers; designing proper hospital work-flow schemes; and promoting adequate sleep, exercise, mental health and time and stress management programs for physicians [18].

## Figures and Tables

**Table 1:** Number of physicians, patient visits and inpatient admissions in Chinese health institutions, 1998–2016.

Year	Number of Physicians (in Millions)	Number of Patient Visits (in Billions)	Number of Inpatients Admissions (in Millions)
1998	2.0	2.1	50.0
1999	2.0	2.1	50.7
2000	2.1	2.1	53.0
2001	2.1	2.1	54.6
2002	1.8 ^a^	2.1	59.9
2003	1.9	2.1	60.9
2004	1.9	2.2	66.7
2005	2.0	2.3	71.8
2006	2.1	2.4	79.0
2007	2.1	3.3	98.3
2008	2.2	3.5	114.8
2009	2.3	5.5	132.6
2010	2.4	5.8	141.7
2011	2.5	6.3	153.0
2012	2.6	6.9	178.6
2013	2.8	7.3	192.2
2014	2.9	7.6	204.4
2015	3.0	7.7	210.5
2016	3.2	7.9	227.3
Percent Change ^b^, %	60	276	355

Footnotes: ^a^ The calculation of physicians present in China has changed slightly since 2002 due to a change in relevant regulations. Data trends are similar despite the change in calculation. ^b^ The percent change in all three indicators was calculated as “(number in 2016–number in 1998)/number in 1998 * 100%”.
